# Abingdon Academy of Medicine

**Published:** 1873-06

**Authors:** 


*Abingdon Academy of Medicine.*

We are pleased to learn, through Dr. W. F. Barr, one of our associates, of the prosperity of this Society. The Abingdon Academy of Medicine is an auxiliary to the Medical Society of Virginia. Our friends of Abingdon are active and accomplished medical men, and we trust our readers may hear from them often.

